# Developmental timing in plants

**DOI:** 10.1038/s41467-024-46941-1

**Published:** 2024-03-27

**Authors:** Enrico Coen, Przemyslaw Prusinkiewicz

**Affiliations:** 1 Department of Cell and Developmental Biology, John Innes Centre, Norwich Research Park, Colney Lane, Norwich, NR4 7UH UK; 2 Department of Computer Science, University of Calgary, 2500 University Dr. N.W., Calgary, AB T2N 1N4 Canada

**Keywords:** Morphogenesis, Plant development

## Abstract

Plants exhibit reproducible timing of developmental events at multiple scales, from switches in cell identity to maturation of the whole plant. Control of developmental timing likely evolved for similar reasons that humans invented clocks: to coordinate events. However, whereas clocks are designed to run independently of conditions, plant developmental timing is strongly dependent on growth and environment. Using simplified models to convey key concepts, we review how growth-dependent and inherent timing mechanisms interact with the environment to control cyclical and progressive developmental transitions in plants.

## Abstract (web summary)

Plants exhibit reproducible timing of developmental events at multiple scales, from switches in cell identity to maturation of the whole plant. Using simplified models to convey key concepts, we review how timing mechanisms interact with the environment to control cyclical and progressive developmental transitions.

## Introduction

Sow the seed of a flowering plant and after a while it germinates. Wait a little longer and an embryonic shoot emerges. At its tip, a microscopic translucent group of cells, the shoot apical meristem, produces leaf primordia on its flanks to a defined beat. Each leaf progresses through a series of stages. As it matures, its cells stop dividing and adopt diverse differentiation schedules to form stomata, veins, pavement cells, hairs. From the leaf axils, additional meristems arise, producing side branches that may reiterate the development of the main axis. Below ground, root apical meristems follow their own developmental time course, producing lateral roots with a regular rhythm as they wind their way downwards. The plant passes through a juvenile phase with characteristic leaf morphologies, an adult vegetative phase and then a reproductive phase, when floral meristems arise, each producing a sequence of organs, ending with those that produce the male and female gametophytes: pollen and embryo sac. Their sperm and egg cells find partners to form zygotes, sporophytes, which develop into embryos within a seed; and so the temporal pageant repeats itself. Non-flowering plants follow similar time schedules, with the sporophyte or gametophyte phases dominating to varying extents.

What determines the orderly and reproducible timing of plant development within each life cycle? It is tempting to conclude that plants contain timers, analogous to clocks, that ensure that developmental programs are switched on at the appropriate point. Yet this analogy with clocks can be misleading.

Clocks are based on counting regular cyclical motions: earth orbiting the sun or spinning on its axis, pendulum swings, or vibrations of a quartz crystal. Relativistic effects aside, time is assumed to progress in the same way whatever the conditions and wherever you are, though we may adjust our clocks according to time zones. Clocks are useful because they help coordinate events: meeting someone, catching a train. Such coordination depends on synchronising clocks according to a standard to ensure time is measured in the same way by all. A clock that runs at different rates according to environmental conditions (e.g., temperature) or spatial context would be considered defective.

Controlled developmental timing was likely selected for during plant evolution for similar reasons that humans invented clocks: to coordinate events. If a seed germinates at an unfavourable time of year, it may not prosper; or if a plant flowers when others are vegetative, it will be unable to cross-fertilise. Internal temporal coordination is also important to ensure cells grow and differentiate in a coherent manner. However, unlike clocks, plant developmental timing may depend on growth and environmental conditions, such as temperature and light. These dependencies are likely not defects in a timing mechanism, but adaptations that allow plants to grow effectively in a variable environment. Moreover, because plants develop iteratively, timing proceeds in parallel in multiple regions, as though there were many cross-talking time zones within a single organism. The notion of global time, progressing independently of conditions, is therefore too restrictive for understanding plant developmental timing.

To broaden the notion of time, we distinguish between two types of developmental timing mechanism: *Growth-dependent timing* relies on generation of material or space through growth. For example, the timing of primordium initiation depends on space becoming available through growth of an apex. *Inherent timing* depends on molecular systems with characteristic delays that are growth independent. For example, circadian rhythms depend on feedback loops through which molecular concentrations rise and fall with defined dynamics. Growth-dependent and inherent timing can interact with each other and with the environment to control developmental timing.

We also distinguish between *cyclical* and *progressive* changes. Cyclical changes include those of cell division, generation of primordia and circadian rhythms. Progressive changes include transitions from embryonic to vegetative and then to reproductive phase, or the developmental progression of meristematic to differentiated cells. Cyclical changes can be embedded within progressive changes, as with repeated generation of primordia during the progression from embryo to maturity; and progressive changes can be embedded within cyclical changes, as with the vegetative to reproductive transition being repeated every year in a polycarpic perennial.

Here we review how different mechanisms control the timing of developmental processes at different temporal and spatial scales. To help clarify concepts, we present simplified models to illustrate fundamental principles and then consider how they relate to observed experimental data. We begin with the timing of a basic cyclical change: cell division.

## Cyclical timing of cell division

**Fig. 1:**
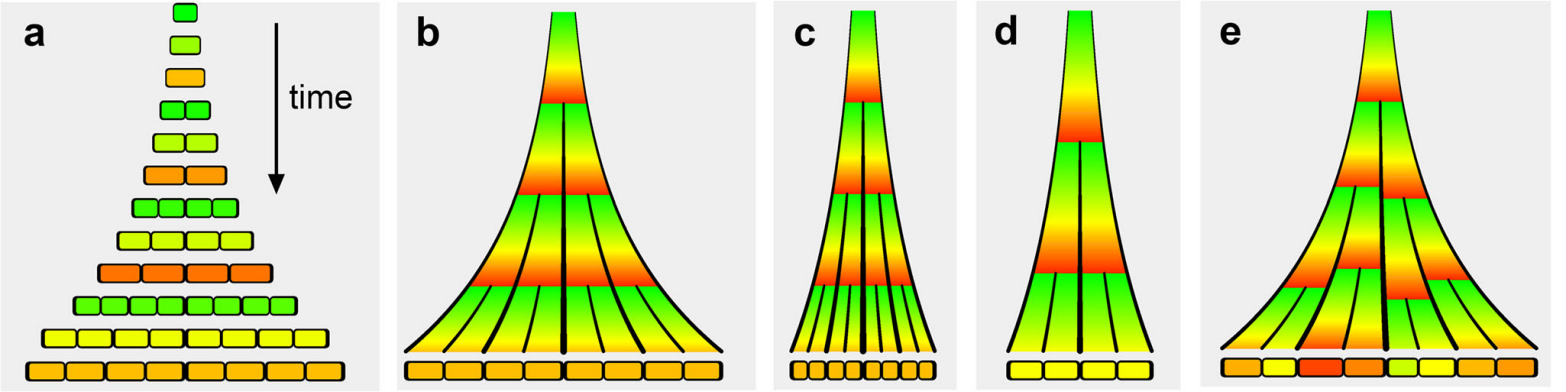
Cell division according to two rules. **a** Cell growing and dividing to form a filament. **b** As (**a**) but shown as a continuum, with a snapshot of cells in their final state at the bottom. **c**, **d** Effect of reducing growth rate. If cells divide with a fixed cycle duration (**c**), cell size decreases with time and final cell number remains unchanged. If a cell divides at a fixed threshold size (**d**), cell cycle duration increases and final cell number decreases. **e** Growth-dependent timing with random displacement of division wall respect to the middle of the cell in the range $\pm$7.5%. Such random displacement desynchronizes divisions and produces cells at the end with diverse cell cycle phases (different colours) and sizes (though sizes remain within a factor of two of each other).

Consider a cell growing and dividing in one dimension to produce a filament (Fig. 1a). The time axis is represented with a downward arrow, and we align stages according to the filament centre, with tissue shown growing out to either side. Rather than discrete snapshots, we may also represent the process in continuous time^1^, with walls of increasing thickness tracing curves as they are displaced by growth (Fig. 1b). Between divisions, cells progress through cell cycle phases, indicated by a colour scale. If all cells grow at a constant growth rate (constant percentage increase in length per unit time) and divide in perfect synchrony, filament length and cell number increase exponentially.

Timing of division could be explained in two ways. One is that cells have an inherent molecular timer. Alternatively, divisions could occur when a cell reaches a critical size. With the first, “timer” mechanism, lower growth rate leads to reduced cell size because cells grow less during the fixed period between divisions (Fig. 1c). By contrast, with the “sizer” mechanism, lower growth rate leads to increase in cell cycle duration, because it takes longer to reach the threshold division size (Fig. 1d). Thus, timing of division for the sizer mechanism is growth dependent, whereas for the timer mechanism it is not. With growth-dependent timing, stochastic asymmetries in division wall position leads to desynchronisation, because unequally sized sister cells reach the threshold size for division at slightly different times (Fig. 1e). Desynchronisation may also arise with inherent timing through a noisy timer, though this may also lead to amplifying fluctuations in cell size (i.e., poor cell size homeostasis)^2^.

There has been a longstanding debate over whether sizer or timer mechanisms underlie cell division control. Much of the plant data supports a sizer mechanism in which the threshold size for cell division can vary depending on internal and external conditions^3^. Consistent with the sizer mechanism, and confirming earlier studies^4^, tracking of divisions in *Arabidopsis* inflorescence meristems has shown that size variability caused by asymmetric division is reduced by larger daughter cells dividing sooner than smaller ones^5^. Division timing is hypothesized to depend on growth-dependent dilution of Kip-related proteins, which inhibit the G_1_-S transition. Cells are born with comparable numbers of these inhibitory proteins, which associate with mitotic chromosomes. Larger daughter cells therefore have a lower initial concentration of inhibitory proteins, and thus reach the permissive threshold for the G1-S transition earlier than their smaller sisters. This process is expected to desynchronise divisions (Fig. 1e), consistent with observed division patterns in plant meristems^6–8^.

Based on the above findings, and in the interests of simplicity, we assume that a growth-dependent timing (i.e., sizer) mechanism controls cell division in what follows. We next consider how such division timing is integrated within progressive developmental timing of the primary growth regions of plants: indeterminate meristems.

## Progressive timing in indeterminate meristems

Apical shoot and root meristems provide the primary source of cells in a growing plant. Cells leaving the slow-growing apical domain undergo developmental switches as they enter other domains. Vascular meristems provide further radial and circumferential growth: cells leaving the cambial domain on one side become phloem and on the other become xylem. Both apical and vascular meristems have the potential to self-maintain indefinitely. How is the timing of developmental transitions of such indeterminate meristems controlled?

## Timing and position

Developmental timing within meristems is intimately connected with the mechanism of spatial patterning^9^. Some aspects of spatial patterning can be controlled by transfer of information from the parent cell to its descendants, while others are explained by transfer of information between coexisting cells^10,11^. Transfer between coexisting cells can provide positional information, a notion first proposed in relation to the French flag problem^12,13^: how can a pattern with constant proportions be generated, irrespective of the overall size of the region being patterned? The problem relates to embryological regulation in animals: if material is removed from an early animal embryo, a normally ordered and proportioned whole-body pattern is achieved with the remaining material. The proposed solution, experimentally supported in several cases^13^, is that cells interpret the concentration of a diffusible morphogen, providing them with positional information with respect to both ends of the tissue. To what extent does the concept of positional information apply to an indeterminate meristem?

**Fig. 2:**
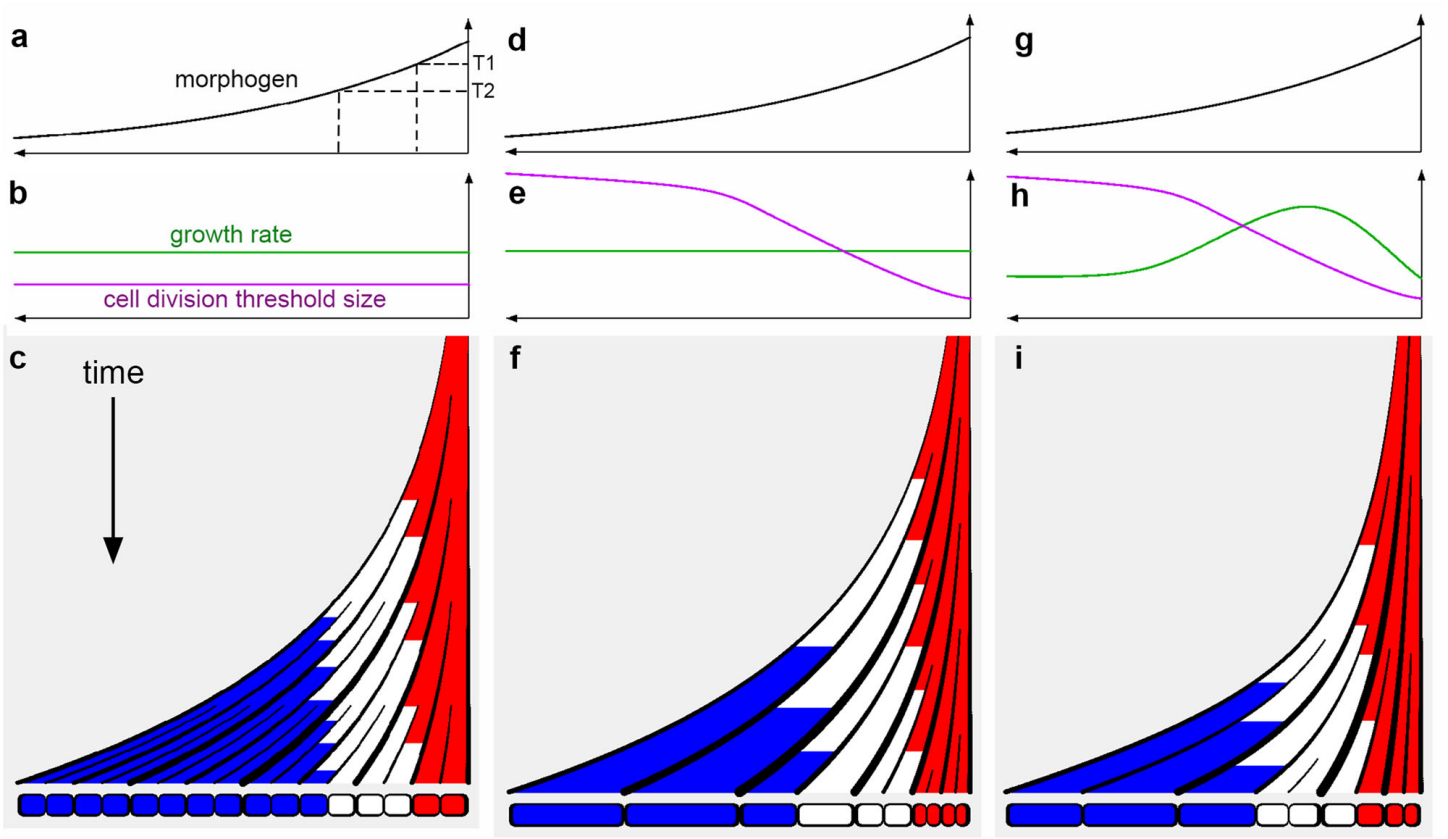
Schematic growth and patterning of an indeterminate meristem, illustrated by a one-dimensional filament. **a**–**c** Uniform tissue growth rate and threshold size for division. **a** Morphogen generated at the right end (apex) forms a gradient. Above threshold T1, cells adopt red identity, between T1 and T2, white identity, and below T2 blue identity. **b** Tissue growth rate (green line) and size at which cells divide (magenta), are both constant. **c** Fate of cells generated by a cell or cells at the right end of the filament shown continuously over time, with final cell pattern shown at the bottom. Red and white domains maintain the same size (domain size homeostasis) while the blue domain increases in size over time. Wall thickness increases with time and cells have uniform sizes. **d**–**f** Same as (**a**–**c**) but with threshold size for division modulated by morphogen concentration to give a rising curve. Cell size increases from right to left, with blue cells no longer dividing as they never reach the increased size threshold. **g**–**i** Same as (**d**–**f**), but with tissue growth rate modulated by morphogen concentration, with the highest rate at an intermediate concentration. The result of this combination of growth rate and cell division threshold curves is slow-growing small cells in the red domain, faster growing larger cells in the white domain, and slow-growing large cells in the blue domain.

Consider a long filament of cells growing uniformly, with a diffusible morphogen generated at the right end. If the filament is approximated as a one-dimensional homogeneous medium, and morphogen decays at a constant rate in each cell, the steady-state distribution of morphogen concentration corresponds to an exponential curve (Fig. 2a). We assume all cells grow at the same rate (green line Fig. 2b), which is slow relative to diffusion, and divide when they reach a fixed threshold size (magenta line, Fig. 2b). We align the filament according to its right end, corresponding to the apex tip, which therefore traces a vertical line over time. The filament cells produced by the apex, or resulting from subsequent divisions, are displaced to the left relative to this line through growth (Fig. 2c; only the lineage derived from one initial apical cell is shown). In accordance with the traditional solution to the French flag problem, cells adopt different identities according to morphogen concentration: above threshold T1 they have red identity, between T1 and T2 they have white identity, and below T2 they have blue identity, generating a French flag pattern of descendants (Fig. 2c).

With this system, morphogen concentration can inform cells of their position only with respect to the right end of the filament, not the distant left end. Unlike the traditional French flag model, proportions are continually changing: once initiated, the blue domain continues to increase in size, while the white and red domains maintain an approximately constant size. We may distinguish between tissue growth and domain growth. Tissue growth is constant throughout the filament, but domain growth becomes restricted to the blue region. The red and white domains thus exhibit size homeostasis despite growth of their component cells.

A consequence of this arrangement is that cell state changes over time: a cell in the red state may transition to white and then blue as it is displaced away from the morphogen source through tissue growth. That is, developmental timing of cell transitions is growth dependent.

## Dual control of growth and cell division

In our simplified scheme, cell size and growth rate do not vary along the length of the filament.  By contrast, indeterminate apical meristems exhibit variation in both. For example, cell size increases with distance from the root tip and growth and division rates peak at defined distances from the tip^14,15^. We may capture variation in cell size by modifying threshold size at which cells divide as a function of morphogen concentration (magenta line, Fig. 2e). The result is a gradient of cell sizes, with smaller cells near the red end and progressively larger cells further away (Fig. 2f). In this example, cells in the blue domain never reach the division threshold, and thus division is arrested.

Incorporating variation in growth rate has been hampered by confusion over whether growth drives division or division drives growth. From a mechanistic perspective, growth rate depends on the extent to which cell walls yield to turgor^16^. The notion that division drives growth implies that insertion of a new division wall leads to an increase in turgor or parental wall-yielding capability, for which there is little experimental evidence. It is therefore unlikely the division drives growth. Instead, either division occurs independently of growth (i.e., timer mechanism), or growth drives division by determining when the cell division threshold size is reached (sizer mechanism). Assuming the sizer mechanism applies, a dual control hypothesis can account for tissue-wide variation in both growth rates and cell size^17–19^. To illustrate this hypothesis, suppose that in addition to modulating the threshold size of division, morphogen concentration influences growth, such that growth rate is highest at intermediate concentrations (green curve, Fig. 2h). This dual effect of the morphogen leads to slow-growing small cells in the red domain, faster growing larger cells in the white domain, and slow-growing large cells in the blue domain (Fig. 2i). Thus, both cell divisions and developmental transitions can exhibit growth-dependent timing, but this timing can be modulated separately. Candidate morphogens controlling transitions in the root are auxin and cytokinin^20,21^. The above example illustrates how both growth rate and division threshold may be controlled along the longitudinal axis of a tissue, but similar principles can account for variation along other axes, such as the radial axis of a root or stem.

## Apical and vascular meristems

We have given a simplified view of how developmental timing may be determined for a filament growing in one dimension. Extending this view to a three-dimensional apex raises the problem that a domed apex has no discrete end from which morphogen can be produced. In ferns and mosses an end is effectively established by an apical cell, which maintains apical identity through asymmetric division and organises growth around it^22^. Angiosperms lack an apical cell but establish a central domain of cells, termed the central zone for shoot apical meristems, which can maintain its size. Size homeostasis of the central domain depends on activity of *CLAVATA* genes, likely through a reaction-diffusion type mechanism^23–28^. The central domain can be considered as equivalent of the red domain in Fig. 2 and may produce morphogens that pattern domains around it.

Developmental transitions in vascular meristems depends on interaction between the *CLAVATA3/ESR-RELATED 41* (*CLE41*) secreted peptide, produced in the phloem, and plasma membrane-bound receptor-like kinase *PHLOEM INTERCALATED WITH XYLEM* (*PXY*), expressed in cambium^29^. CLE41 peptide has been proposed to act as a morphogen that specifies phloem, cambial or xylem cell types, depending on morphogen concentration and cell size^30^. In terms of our simplified scheme, phloem can be considered a morphogen-producing red domain. In this case, however, cell divisions would be restricted to the white (cambial) domain because the threshold size for division is high in both the red (phloem) and blue (xylem) domains (i.e., the magenta curve for cell size division threshold is U-shaped). Keeping our view aligned with the white cambial domain, cells exiting to the left would adopt blue (xylem) identity, whereas cells exiting to the right adopt red (phloem) identity. Both the red and blue domains, and their component cells, enlarge through growth (the red domain enlarges because cells with high morphogen concentration switch to red identity when above a threshold size^30^). By contrast, the white domain exhibits size homeostasis and comprises relatively small cells that continue to divide. Thus, timing of developmental transitions in both apical and vascular meristems is likely growth-dependent, though the control of division and growth can vary.

## Cyclical timing of primordium production

In addition to progressive timing, apical meristems exhibit cyclical timing through regular production of primordia. Two broad mechanisms have been proposed to underlie this cyclical timing: inherent and growth-dependent. To illustrate these mechanisms with our filament, we assume that tissue growth only occurs in the red domain, but this domain does not enlarge because of size homeostasis.

**Fig. 3:**
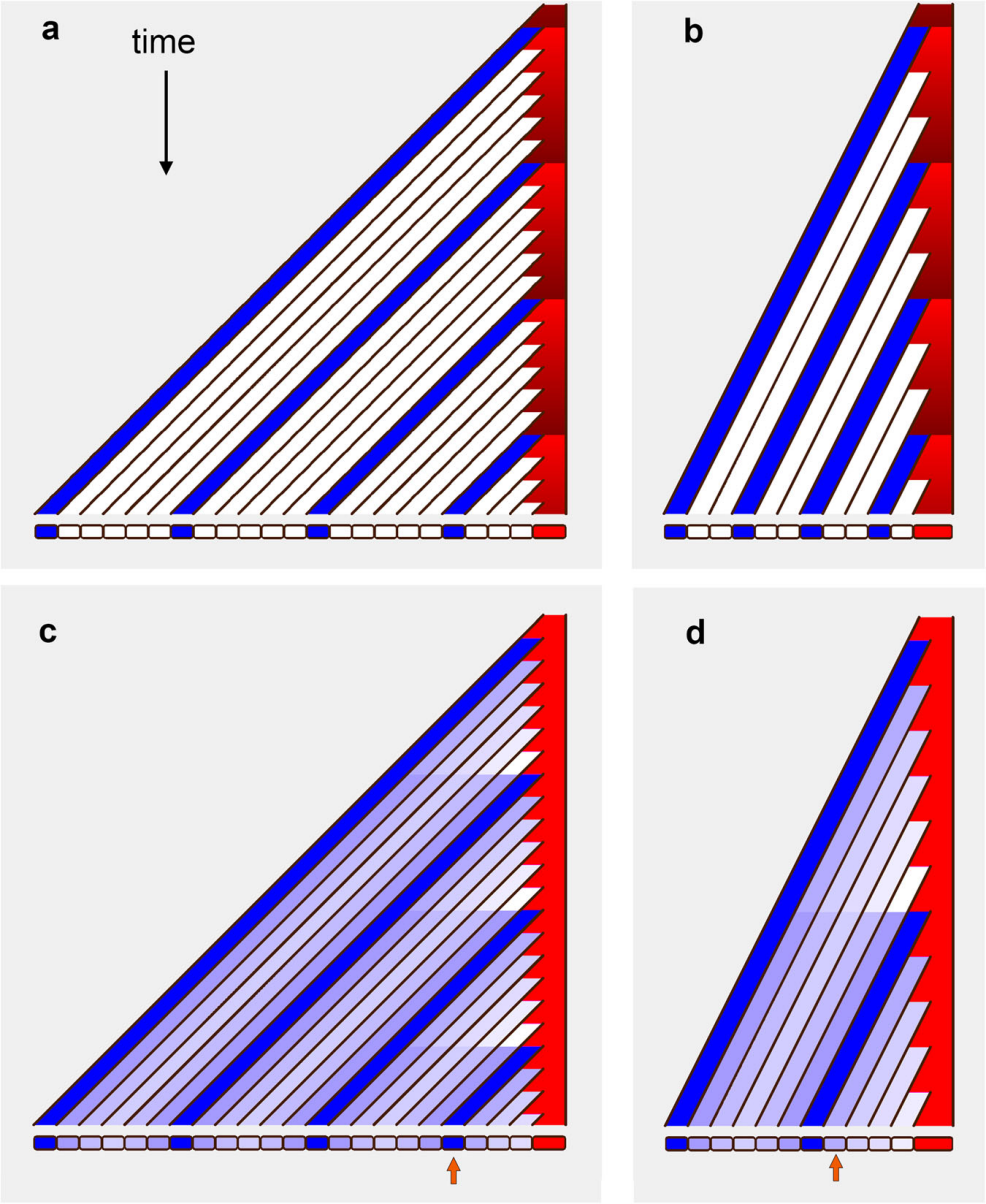
Generation of repeating units through inherent or growth-dependent timing. Only the tissue in the red domain grows. **a** Inherent timing. The red domain cycles between light red and dark red with constant period. Cells adopt white identity when exiting the red domain in the light red state, but blue identity when exiting in the dark red state. Regularly spaced blue domains are generated. **b** As (**a**), but at a slower growth rate. Blue domains are spaced more closely, giving smaller repeating units (similar to the timer mechanism for cell division (Fig. 1c)). **c** Growth-dependent timing. Blue cells produce an inhibitory morphogen that prevents cells exiting the red domain from switching to the blue state. Red-exiting cells switch to white unless the nearest blue cell is sufficiently far away that inhibitory morphogen drops below a critical threshold. Regularly spaced blue domains are generated. **d** As (**c**) with slower growth rate. Fewer blue domains are generated but with the same spacing, giving fewer repeating units of the same size (similar to the sizer mechanism for cell division (Fig. 1d)). In (**c**) and (**d**), identity at a fixed distance from the end (orange arrow) oscillates between white and blue states through cells being displaced by growth, rather than temporal oscillations happening with the same cell, as in the light-dark red cycles in (**a**) and (**b**).

With inherent timing, the red domain changes state over time from a light red to dark red in regular cycles (graded red column, Fig. 3a). If cells exit the red domain in light red state, they adopt white identity; whereas if they exit in the dark red state, they adopt blue identity. Regularly spaced blue domains are generated, with a period dictated by the light-dark red oscillator. Reducing growth rate does not change cycle duration, but leads to smaller repeating units (Fig. 3b), as with the timer mechanism of cell division (Fig. 1c).

To illustrate growth-dependent timing, suppose blue cells maintain a fixed concentration of an inhibitory morphogen that diffuses out (concentration indicated by light blue intensity, Fig. 3c). Cells exiting the red domain adopt a blue state if the concentration of inhibitory morphogen is low, but a white state if the concentration is above a threshold. Consequently, a cell exiting the red domain switches to blue as soon as there is sufficient space between it and the neighbouring blue cell for the inhibitor concentration to fall below threshold. Regularly spaced blue domains are generated. In this case, reducing growth rate does not change the length of the repeating unit, but increases cycle duration (Fig. 3d), as with the sizer mechanism of cell division. If we observe our filament at a fixed distance from the right end (orange arrow, Fig. 3c, d), cell state oscillates over time between the blue and white. This oscillation is not caused by the same cell changing state, as in the dark-light red oscillations of the inherent timing mechanism, but by cells in different states passing the same point in space (relative to the right end) as they are displaced by growth.

An inherent timing mechanism likely underlies somite formation in vertebrates^31–33^. Somites bud off periodically in pairs at the anterior tip of the presomitic mesoderm. The regular timing of somitogenesis has been explained by a clock-and-wavefront model, operating according to the same principles as in Fig. 3a, except that axial extension of the presomitic mesoderm proceeds by cell recruitment rather than growth and division. A critical test of the model was to show that oscillations in gene expression can continue in dissociated neural progenitors^34^.

An inherent timing mechanism has also been proposed for the generation of lateral root primordia^35,36^. Oscillatory expression of genes that mark presumptive lateral root primordia is observed about a millimetre or so from the root tip, and hypothesised to be output of an inherent timing mechanism, or root clock^35^. However, it is unclear whether the observed oscillations involve cells autonomously switching identity, as required by a clock mechanism, or cells with different identities passing the same position in space (relative to the root tip) as they are displaced by growth. The latter assumption has led to an alternative model, based on growth-dependent timing^37,38^.

In contrast to somitogenesis, where repeating units are generated in synchronised pairs, plant primordia can be produced by apical meristems with a variety of timings and spatial arrangements (phyllotaxis). Timing here is embedded in a two-dimensional patterning system, not fully captured by our one-dimensional filament. Models for generating phyllotactic patterns based on an inherent timing mechanism have been proposed^39,40^. However, a growth-dependent mechanism is more widely accepted: primordia arise where and when space for them becomes available within a morphogenetically active zone^41–44^ positioned at the periphery of the central domain of the apical meristem. A growth-dependent mechanism is a more attractive explanation of phyllotaxis because timing and spacing of primordia are automatically coupled, so a slower-growing apex produces primordia at a lower rate. Moreover, underlying molecular mechanisms for such growth-dependent timing have been identified. Rather than primordia producing a diffusible inhibitor shown in Fig. 3c, these mechanisms are based on primordia depleting the concentration of a diffusible promoter of primordium initiation, auxin, in their neighbourhood^45–47^. Local auxin depletion is enhanced by auxin being transported towards primordia through PIN proteins, reinforcing auxin maxima. Questions remain about how PIN localisation, auxin distribution and auxin response is controlled^38,48–51^, and the mechanisms by which additional genetic and molecular factors affect the timing of the patterning process^52^.

**Fig. 4:**
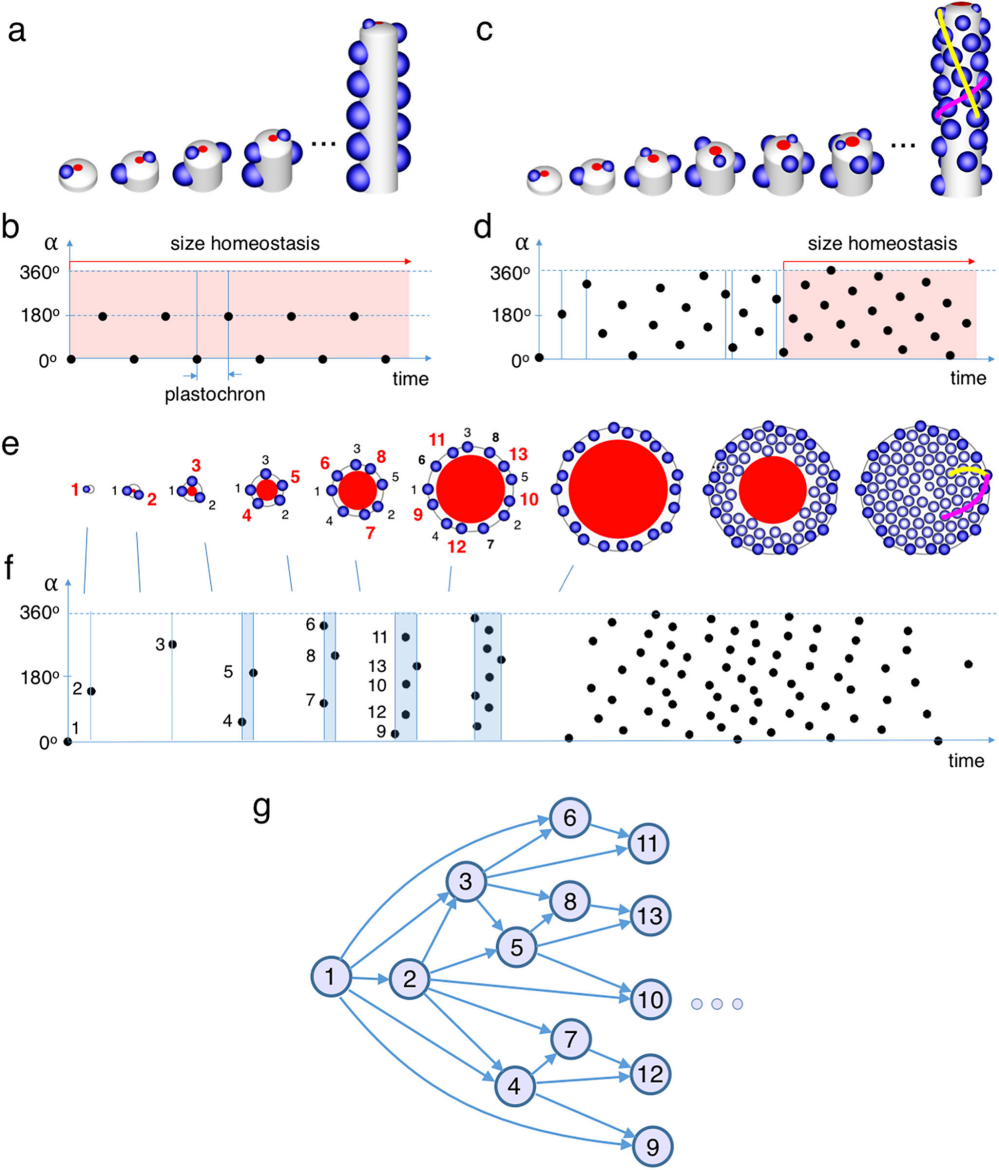
Generation and timing of phyllotactic patterns. **a**, **b** Development of a distichous pattern of primordia (blue) on a growing shoot apical meristem (grey) with a small central domain (red) exhibiting size homeostasis (red shading in (**b**)) throughout. Primordia emerge with a constant plastochron. Angle α indicates the angular position of primordia. **c**, **d** As (**a**, **b**), with radius of the central domain gradually increasing before reaching size homeostasis. A spiral (helical) phyllotactic pattern emerges, with 3 and 5 parastichies running in opposite directions (magenta and yellow, respectively). Plastochron decreases before size homeostasis is reached, and is subject to fluctuations. **e**, **f** As (**a**, **b**), with the meristem size and the central domain initially expanding in concert, and the central domain subsequently contracting. The resulting pattern has 13 and 21 contact parastichies. In the expansion phase, primordia emerge in bursts, shaded blue in (**f**), followed by periods in which newly initiated primordia migrate to positions that are asymmetric with respect to their neighbours. **g** Causal diagram capturing temporal precedence (arrows) between primordia initiation events (circles) necessary for the pattern (**e**) to emerge. Events not connected by directed paths (e.g., the initiation of primordia (3,4) or (6,8)), are not causally related and can take place in any order.

The above growth-dependent timing mechanism can generate primordia in different spatiotemporal modes, depending on the geometry and growth of the meristem, and the dynamics of the central and active zones^53,54^. If the central domain (red in Fig. 4) exhibits size homeostasis and is relatively small, primordia emerge sequentially on opposite sides of the surrounding active zone, producing a distichous phyllotactic pattern (Fig. 4a). This pattern emerges because a new primordium can only initiate when the previous primordium has been displaced sufficiently far away through tissue growth. With constant tissue growth rates, the time interval (plastochron) between successive primordia is constant, and the angular position of primordia, α, alternates between 180^o^ and 0^o^ (Fig. 4b). Reducing growth rate would increase plastochron, as with our hypothetical filament (Fig. 3d).

If the central domain gradually increases in size, while the size of initiated primordia remains constant, space available for a new primordium becomes determined not by the previous primordium alone, but by the last two primordia. With a further size increase, space for a new primordium is no longer defined by its two immediate predecessors, but by pairs (occasionally, triplets) of primordia initiated even earlier. This process typically leads to the formation of a spiral phyllotactic pattern with the numbers of conspicuous spirals (contact parastichies) progressing according to Fibonacci sequence: 1, 2, 3, 5, 8, 13,…. In shoots of many plants, this progression ends when the central domain attains size homeostasis (red shading, Fig. 4c) and meristem geometry reaches steady state. For example, Fig. 4c shows a progression resulting in conspicuous spirals (contact parastichies): three running in one direction (one of them shown with magenta line) and five running in the opposite direction (yellow line) in the steady state, as observed in the flowering shoot of *Arabidopsis*^55^. Throughout the patterning process, plastochron may systematically change over time (Fig. 4d) while also being subject to smaller, random variation^52,56^.

A qualitatively different situation occurs if the central domain does not exhibit size homeostasis but grows at a rate commensurate with tissue growth, while meristem geometry is not in steady state. This dynamics is found in early developmental stages of flower heads, such as those of sunflower and *Gerbera*^57^. Circumferential growth of the active zone could lead to periodic doubling of primordium numbers, with sets of new primordia arising between older ones in a geometric progression: 1, 2, 4, 8, 16… in an analogy with the insertion of new walls in synchronised cell division (Fig. 1b). In composite inflorescence heads, however, an additional factor intervenes: newly initiated primordia, as detected by auxin concentration maxima, migrate towards their oldest near neighbour (the second primordium is an exception: its migration direction appears to be random). The resulting asymmetry leads to primordia arising in bursts (Fig. 4e, Supplementary Movie 1, and Fig. 4f, blue shading), with the number per burst increasing according to the Fibonacci sequence^57^.

Temporal coordination between initiation events can be captured by a causal diagram^58^, similar to an ontogenetic graph^44^: a (semi) lattice in which each arrow indicates a causal interaction between primordia (Fig. 4g). Events not connected by a directed path of arrows are concurrent and can take place in any order without affecting the result^59^. A linear time axis (Fig. 4f) imposes a total order on all events, whereas a causal diagram only defines a partial order. Linear time is convenient when recording experimental observations or results from individual runs of simulations, but a lattice description of time is advantageous when characterizing and analysing underlying mechanisms. Its usefulness is not limited to inflorescences but extends to all processes in which the exact sequence of events is not critical.

Expansion of the central domain, as revealed by *CLAVATA3* expression, characterises the early stages of flower head development^57^. At later stages the central domain contracts, propagating the pattern towards the centre. In both phases, the uniform growth/contraction of the radially symmetric central domain coordinates and partially synchronizes the initiation of primordia, resulting in the formation of a regular pattern (Fig. 4g) without imposing a linear order on initiation events.

## Progressive timing in determinate meristems

If a plant comprised solely indeterminate meristems which cyclically produced further indeterminate meristems, it would generate evermore highly branched axes. Such proliferation is prevented in two ways. First, growth of some axes is inhibited, through mechanisms such as apical dominance. Second, some meristems adopt a determinate rather than indeterminate growth pattern. Determinate meristems have a limited duration of growth and can be of two types: primordia that produce organs of limited size, such as leaves or petals; and meristems that produce a limited number of organs before arresting, such as floral meristems.

Determinate meristems typically follow a progressive developmental time course after their initiation. We use the term “meristem age” to refer to how far the meristem is along this time course. Thus, a newly initiated primordium or meristem has zero meristem age, and age then increases in a growth-dependent and/or inherent time-dependent manner.

## Organ primordia

Young organ primordia share features of indeterminate meristems, such as domains exhibiting size homeostasis. *Arabidopsis* leaf primordia, for example, have a proximal domain at the lamina base with high growth and division rates^60,61^. This domain can be most clearly seen in mutants for *SPEECHLESS* (*SPCH*), which lack stomata^19^. The proximal domain maintains its length during early development, likely through a morphogen generated from the leaf base^19,61^.

As leaf cells exit the proximal domain, many of them cease dividing, enlarge and differentiate, leading to a gradient of increasing cell size towards the leaf tip. The onset of differentiation may be defined by when cell division arrests, though DNA replication may continue, leading to endoreduplication. Thus, at early developmental stages,  potential for differentiation onset may depend on when cells exit the proximal domain (i.e., growth-dependent timing). Timing of differentiation onset is further modulated by cell type. *SPEECHLESS* promotes stomatal precursor divisions, both within and outside the proximal domain, thus potentially delaying differentiation onset^19^. These divisions are asymmetric, producing progressively smaller stomatal precursors^62^, perhaps because the threshold size for division is not fixed but is a fraction of birth size. When cells fall below a threshold size, they become more likely to differentiate as stomata^63^. Thus, commitment to stomatal differentiation has been hypothesised to depend on cells reaching a minimal threshold size.

Following division, cells follow a progressive differentiation time-course, ending with mature, fully differentiated cell types. Timing of some cell shape changes, such as formation of increasing convoluted pavement cell outlines, is likely growth-dependent^64^. Other cell types, such as stomata and trichomes, follow distinct cell-autonomous growth patterns, creating local heterogeneity in cellular growth rates^65^.

Except for some species of *Streptocarpus*, in which growth and cell division continue indefinitely near the leaf base^66,67^, cells in the proximal domain eventually cease to divide and begin to differentiate. In *Arabidopsis*, proximal division arrest is evident in *speechless* mutants^19^ and may occur later for *SPCH-*dependent (stomatal) divisions. Growth rate also declines throughout the leaf, eventually dropping to zero. From a mechanical perspective, growth arrest arises when cell walls no longer undergo creep in response to turgor, because of increase in wall thickness, and/or increase in yield threshold^16^.

The timing of growth slowdown and arrest, and thus final leaf size, depends on genotype and environment^68^. If timing was controlled by growth slowing down as leaves approach an upper size limit, a decrease in growth rate should be compensated for by an increase in growth duration, to give a similar final size. Such compensatory effects are observed in *Arabidopsis* accessions subject to water deficit or shading^69,70^. However, a water deficit in sunflower reduces growth rate without affecting growth duration, yielding smaller leaves^71^. Water deficit in this case causes cell division arrest at a smaller leaf size, indicating that cell size may play a role in limiting final leaf size. Lack of stomata also causes division arrest at a smaller-than-usual leaf size, likely through physiological impairment^19^. Thus, diverse mechanisms may contribute to growth arrest but how they interact with each other, and the environment remains unclear.

Developmental timing in determinate organs varies between species and organ types. In compound leaves, differentiation may be delayed during the generation of multiple leaflets^72^. In some peltate leaves, such as those of Nasturtium, an early lobed shape can become rounded through later differential growth^73^. In many perennials, growth rates and cell sizes are distributed uniformly over the maturing leaf rather than showing a strong proximodistal pattern^74^. In petals, cell divisions can continue throughout the organ until late developmental stages^75^. The variation in timing of division arrest and differentiation between species and organ types may represent adaptations to diverse ecological constraints, such as coordination of flower opening or resistance to herbivores^76^.

## Floral meristems

Floral meristems generate a limited number of organs in a concentric arrangement: typically sepals, petals, stamens and finally carpels. Floral meristem termination usually occurs after carpel initiation and depends on activation of C-function organ identity gene. In *Arabidopsis*, the C-function gene *AGAMOUS* (*AG*) promotes termination by repressing meristem maintenance gene *WUSCHEL* via transcription factors *KNUCKLES* (*KNU*)^77–81^. It has been proposed that activation of *KNU* by *AG* requires about two rounds of cell division to dilute repressive chromatin marks^80,81^, suggesting growth-dependent timing. Activation of C-function genes in turn depends on floral meristem identity genes, such as *FLORICAULA/LEAFY* ^82,83^. Thus, initiation of floral meristem identity sets off a train of events leading to meristem arrest. The delay between floral meristem initiation and termination varies between species^84^, though the extent to which timing is growth-dependent and/or inherent is unclear.

If timing of sepal primordium initiation becomes variable, as in mutants of *DEVELOPMENT RELATED MYB-LIKE* *1* of *Arabidopsis*, differences in primordium size lead to corresponding differences in organ size in the mature flower^85^. Thus, a later-initiating sepal primordium will generate a smaller mature sepal. In this case, timing of growth arrest is not local to each primordium but depends on aging of the entire flower.

## Progressive timing over the plant life cycle

Following germination, the main shoot of a flowering plant exhibits progressive changes, evident from the types of leaf, branch, and internode it produces. For example, early rosette leaves of *Arabidopsis* are round, with few serrations, whereas later leaves are more elongated and serrated. This progressive change in morphology is termed the juvenile-to-adult transition, or vegetative phase change^86^. A second transition is from vegetative to reproductive, which leads to inflorescence and flower production. The timing of these transitions depends on environmental cues and age^87^. The notion of age is not straightforward, however, because plants initiate multiple meristems at different times. It is therefore useful to distinguish between plant age, which refers to age of the entire plant since germination, and meristem age, which refers to the age of a meristem since its initiation. These ages are similar for the main apical meristem, but meristem age is less than plant age for laterals.

## Vegetative phase change

The juvenile-to-adult transition is mediated through plant age-dependent decline in levels of the microRNA, miR156, in the apex and leaf primordia^86,88^. miR156 represses SQUAMOSA PROMOTER BINDING PROTEIN-LIKE (SPL) transcription factors which promote adult traits. It was originally thought that changes in miR156 expression mediated control of vegetative-to-reproductive as well as the juvenile-to-adult transition and thus constituted the plant age pathway^89^. However, later studies showed that competence to flower, while plant age-dependent, can be controlled independently of miR156^90^. We propose that the response controlled by miR156 be termed “maturity” as it relates specifically to the juvenile-to-adult transition. Decline in miR156 expression thus leads to increase in maturity. Changes in leaf morphology during vegetative phase change can be explained through interactions between maturity (dependent on plant age) and developmental progression of each leaf since its initiation (dependent on leaf meristem age). These interactions are mediated through the effects of miR156 target *SPL9* on *CyclinD3*, which can influence both growth rates and cell division thresholds^91^.

In polycarpic perennials the vegetative-to-reproductive transition is repeated annually within the same plant. This growth habit requires a proportion of meristems to remain vegetative when the plant is flowering. In *Arabis alpina*, young laterals (i.e., with low meristem age) are less mature than their parental apex, based on expression of the miR156 target, *SPL15* ^92,93^. This dependency of maturity on meristem age allows some regions of the plant to remain vegetative by not responding to vernalisation cues, thus enabling the perennial habit^93^.

## Timing of transition to floral identity

The vegetative-to-reproductive transition is typically considered at the level of the entire plant, with the plant assigned a single flowering time. However, meristems on the same plant undergo transitions to floral identity at different times. The control of such timing can be described through a continuous abstract variable, vegetativeness (*veg*), which denotes how far a meristem state is from floral identity^94^. To illustrate the usefulness of this concept, we begin by considering transitions of the inflorescence apex in *Arabidopsis* (i.e., the apex that will generate branches and flowers above the vegetative rosette).

**Fig. 5:**
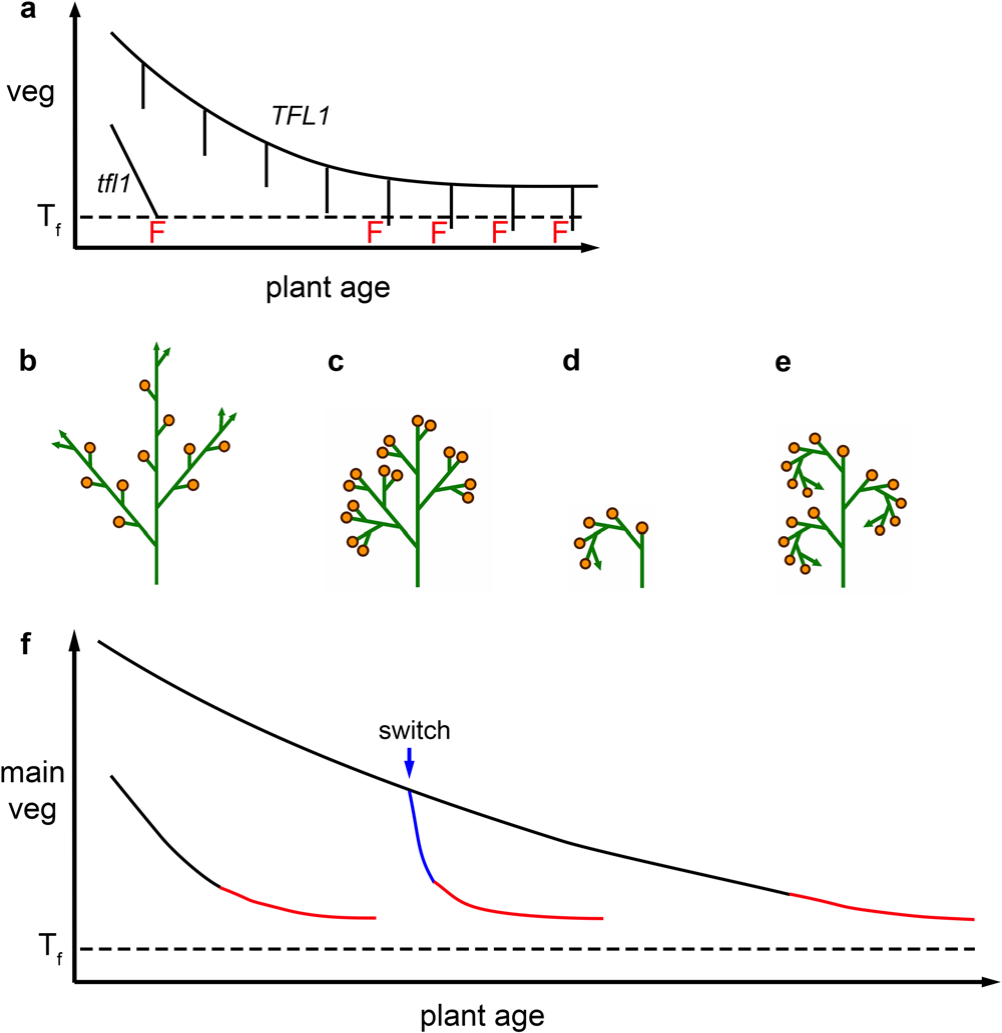
Control of timing of transition to floral identity through changes in vegetativeness. **a** The model. In a *tfl1* mutant inflorescence, *veg* in the main apex declines rapidly and reaches the floral threshold *T*_*f*_ leading to production of a terminal flower. In wild type (*TFL1*), *veg* starts off higher and declines more slowly in the main apex, while *veg* drops in young lateral meristems (vertical lines). This drop is transient unless it reaches the floral threshold, in which case the meristem switches to floral identity. **b**–**e** Examples of inflorescences generated by the model with different parameter values: **b**, raceme; **c**, panicle; **d**, cyme; **e**, thyrse. **f** Incorporating environmental controls of flowering time. In early-flowering plants (lower black curve) *veg* in the main apex approaches floral threshold earlier than in late-flowering plants (upper black curve), leading to generation of a flowering raceme (red line). If late-flowering plants are moved to an environment that induces flowering, *veg* in the main apex drops (blue line).

In *terminal flower 1* (*tfl1*) mutants of *Arabidopsis* the inflorescence rapidly terminates with formation of a flower. The timing of the floral transition can be explained by *veg* in the main inflorescence apex declining with plant age (downward sloping *tfl1* line, Fig. 5a) until it reaches the floral threshold (dotted black line), whereupon the apex adopts floral identity. By contrast, in wild type, the main apex and first lateral branches (coflorescences) remain indeterminate and floral identity is only adopted by later laterals, giving a raceme (Fig. 5b). This architecture can be explained by differential modulation of *veg* in laterals compared to the main apex. *TFL1* is expressed in the main inflorescence apex^95^, where it raises *veg*, preventing the apex from reaching the floral threshold (curved *TFL1* line, Fig. 5b). However, *TFL1* is not expressed in young lateral meristems, causing their *veg* to drop (vertical lines, Fig. 5a). In the first lateral meristems, the drop may not take *veg* down to the floral threshold, and *veg* returns to its parental value, causing an indeterminate branch to be produced. In subsequent laterals, *veg* reaches the threshold, activating floral identity. This timing mechanism underlies models of wild-type and mutant Arabidopsis architectures^94,96^.

An attractive feature of this mechanism is that it can account for a range of inflorescence architectures through simple parameter changes^94^. In some species, flowers terminate multiple branches to form panicles (Fig. 5c), which can be explained by *veg* in laterals being the same as in their parental apex (i.e., *veg* level only depends on plant age). In other species, flowers terminate the main axis and successive branches to form cymes (Fig. 5d) or thyrses (Fig. 5e^97^,), which can be explained through the inverse of the raceme-generating mechanism: the difference between *veg* and the threshold in young laterals is greater instead of lower, compared to the parental apex. Thus, as with vegetative phase change, varying the response to plant age and/or meristem age can modulate timing of the reproductive (floral) transition, leading to diverse growth strategies.

## Environmental coordination

The time at which plants initiate inflorescence development depends on seasonal cues, such as photoperiod and temperature. We may illustrate differences in timing through curves of *veg* decline in the main apex with plant age (Fig. 5f). The red region of the curve is when lateral floral meristems are produced (e.g., the raceme). In late-flowering plants, such as those grown in non-inductive conditions, initial *veg* levels are high and/or rates of *veg* decline are low (upper black curve); whereas in early-flowering plants, initial *veg* levels are low and/or rates of *veg* decline are high (lower black curve). A switch in environmental conditions that induces flowering corresponds to steepening of the *veg* decline (blue curve, Fig. 5f).

The mechanism whereby photoperiod modifies flowering has been intensively studied in *Arabidopsis*^98–100^. Numerous transcripts in *Arabidopsis* exhibit daily oscillations in expression, controlled by a circadian clock (inherent timing), entrained by light/dark cycles^101^. Among these transcripts is that of *CONSTANS* (*CO*), which encodes a transcription factor that requires light for nuclear stabilisation. During the short days of winter, *CO* expression peaks at dusk. In the absence of light, CO is therefore destabilised. However, as days lengthen in spring, the *CO* expression peak becomes coincident with evening light, allowing CO stabilization. CO then activates *FLOWERING LOCUS T* (*FT*) expression in leaf vascular tissue, whereupon FT protein travels to meristems^102^ and promotes flowering (by reducing *veg* of laterals in our scheme). The ability of plants to respond to photoperiod depends on plant age, through a mechanism that is independent of vegetative phase change^90^. Thus, above a certain plant age, exposure long days leads to a more rapid *veg* decline in the apex (blue curve, Fig. 5f).

Age-dependent responsiveness may also control anthesis, as illustrated by sunflower inflorescences. Interior sunflower florets are initiated as the central domain contracts^103^ (Fig. 4e). Over a 24 h period, rapid domain contraction can lead to initiation of multiple floret primordia, forming a ring, or pseudowhorl, of meristems born within a 24 h window. As the inflorescence matures, florets above a critical meristem age may become competent to undergo anthesis in response to a light-entrained circadian signal^104^. If floret primordia grow and age concurrently at the same rates, florets reaching competence during a 24 h period will be organised as a pseudowhorl, accounting for the observed daily synchronised emergence of florets in rings, a process which may promote pollination efficiency^104^.

In some *Arabidopsis* ecotypes, exposure to prolonged cold in winter promotes flowering through the process of vernalisation, mediated by the transcription factor encoded by *FLOWERING LOCUS C* (*FLC*)^105^ which represses flowering and thus raises *veg* in our scheme. Cold response is distributed throughout the vernalisation regulatory network, but the long-term cold response depends on the activity of transcription factor NTL8. The NTL8 protein has a long half-life and is produced in meristematic domains that exhibit size homeostasis^106^. Within these domains, the amount of NTL8 protein has been hypothesised to increase in each cell at a constant inherent rate. The amount of NTL8 per cell is halved when it divides. Thus, if tissue growth rate is low, NTL8 is halved less often by cell division, giving a higher mean NTL8 concentration (assuming cells divide at a common threshold size). When temperature drops in winter, NTL8 concentration is therefore enhanced by slow growth. NTL8 activates *VERNALIZATION INSENSITIVE 3* (*VIN3*) that promotes Polycomb-dependent epigenetic silencing of *FLC*^105^. Prolonged NTL8 during winter leads to irreversible silencing of *FLC* in more and more cells. When temperature rises in spring, *FLC* levels are unable to rise because many *FLC* gene copies have been stably silenced. Silencing of *FLC* by prolonged cold therefore reduces *veg*, which accelerates flowering. Thus, timing is growth-dependent (cold-sensing depends on reduced tissue growth rate), and also has an inherent component because the rate of *FLC* silencing is assumed to be constant for a given NTL8 concentration.

## Outlook and future perspectives

Developmental timing in plants is strongly dependent on growth. Growth-dependent timing ensures that developmental transitions are linked to how much space and material is being generated. It thus allows timing to be coupled to general environmental conditions, such as light and temperature, through their effects on growth. Inherent timing interacts with growth-dependent timing and other environmental sensing mechanisms (e.g., responses to light/dark), to allow developmental transitions to be coordinated with diurnal or seasonal changes. A major challenge is to understand the molecular mechanisms underlying these interactions. For example, how is the decline of miR156 expression or *veg* with plant age controlled; and how are growth rates, cell division thresholds and cell differentiation programs regulated and integrated? Answering these questions requires not only identifying relevant genes, but also capturing the dynamics of their interactions.

Another challenge is to clarify how signals with different ranges and propagation rates interact with growth to influence timing. For example, local interactions underlying phyllotaxis lead to partially ordered events in which primordium age decreases up the plant axis or towards the centre of a flower head (acropetal patterns). Nevertheless, further development of these primordia into lateral branches^107^, the transition to flowering^108^, or the opening of flowers^109^ may occur in an order different from bud initiation. This temporal order may be tightly regulated by diverse mechanisms, including long-distance propagation of control signals. The molecular nature and modes of propagation of long-distance signals have been subject of both experimental and theoretical investigation^102,110,111^, but many aspects remain open. For example, different developmental and flowering sequences have been explained through acropetal and/or basipetal control signals propagating within growing inflorescences^112–114^. The finite-speed propagation of these signals in expanding structures produces complex spatiotemporal patterns consistent with those observed experimentally, but whether such mechanisms operate within plants has yet to be established.

A further issue is how developmental timing in plants compares with that in animals. In contrast to plants, animal morphogenesis occurs during more restricted periods of the life cycle, often shielded from environmental fluctuations through parental care. Inherent timing may therefore play greater role in animals than in plants, as illustrated with the control of somitogenesis. However, following somitogenesis, each member of a limb pair attains the same final size through concurrent intrinsic growth^115^, which may involve growth-dependent timing. Embryos of ectotherms (e.g., reptiles, fish or insects) experience a more plant-like exposure to varying environmental conditions during development. Increased incubation temperature typically leads to increased developmental rate and growth rate^116^, which could be explained by growth-dependent timing and/or other temperature-sensitive timing mechanisms. Environmental cues may also modify animal developmental traits such as sex in reptiles and fish^117^ and other polyphenisms^118^. Thus, developmental timing in plants and animals may share many features, though the extent to which different mechanisms contribute varies.

While many fascinating questions have still to be answered, one thing seems clear: developmental timing in plants is distinct from our notion of global clock-based time, progressing independently of conditions. It is nearer to the notion of time described by theoretical physicist Carlo Rovelli: *The single quantity “time” melts in a spiderweb of times. We do not describe how the world evolves in time: we describe how things evolve in local time, and how local times evolve relative to each other. The world is not like a platoon advancing at the pace of a single commander. It’s a network of events affecting each other*^119^.

### Supplementary information

Description of Additional Supplementary Files

Supplementary Movie 1
