# Supplementary material for: Developmental timing in plants
Source: Nat Commun. 2024 Mar 27;15:2674. doi: 10.1038/s41467-024-46941-1 (PMC10965974; doi:10.1038/s41467-024-46941-1)
Supplement: Supplementary file 1 — Description of Additional Supplementary Files [file 41467_2024_46941_MOESM1_ESM.pdf]

<u>Description of Additional Supplementary Files</u>

Supplementary Movie 1: Generation of phyllotactic pattern in a composite inflorescence. Meristem size and the central domain (red) initially expand in concert, leading to initiation of floret primordia (dark blue) in available space. The central domain subsequently contracts, allowing primordia (pale blue) to fill the internal space.
